# Brain-derived neurotrophic factor enhances the excitability of small-diameter trigeminal ganglion neurons projecting to the trigeminal nucleus interpolaris/caudalis transition zone following masseter muscle inflammation

**DOI:** 10.1186/1744-8069-9-49

**Published:** 2013-09-30

**Authors:** Mamoru Takeda, Masayuki Takahashi, Junichi Kitagawa, Takuya Kanazawa, Masanori Nasu, Shigeji Matsumoto

**Affiliations:** 1 Department of Physiology, School of Life Dentistry at Tokyo, Nippon Dental University, 1-9-20, Fujimi-cho, Chiyoda-ku, Tokyo 102-8159, Japan; 2 Research Center of Odontology, School of Life Dentistry at Tokyo, Nippon Dental University, 1-9-20, Fujimi-cho, Chiyoda-ku, Tokyo 102-8159, Japan; 3 Division of Oral Physiology, Department of Oral Biological Sciences, Niigata University Graduate School of Medical and Dental Sciences, 2-5274, Gakkocho-dori, Niigata 951-8514, Japan

## Abstract

**Background:**

The trigeminal subnuclei interpolaris/caudalis transition zones (Vi/Vc) play an important role in orofacial deep pain, however, the role of primary afferent projections to the Vi/Vc remains to be determined. This study investigated the functional significance of hyperalgesia to the brain-derived neurotrophic factor (BDNF)-tyrosine kinase B (trkB) signaling system in trigeminal ganglion (TRG) neurons projecting to the Vi/Vc transition zone following masseter muscle (MM) inflammation.

**Results:**

The escape threshold from mechanical stimulation applied to skin above the inflamed MM was significantly lower than in naïve rats. Fluorogold (FG) labeling was used to identify the TRG neurons innervating the MM, while microbeads (MB) were used to label neurons projecting to the Vi/Vc region. FG/MB-labeled TRG neurons were immunoreactive (IR) for BDNF and trkB. The mean number of BDNF/trkB-IR small/medium-diameter TRG neurons was significantly higher in inflamed rats than in naïve rats. In whole-cell current-clamp experiments, the majority of dissociated small-diameter TRG neurons showed a depolarization response to BDNF that was associated with spike discharge, and the concentration of BDNF that evoked a depolarizing response was significantly lower in the inflamed rats. In addition, the relative number of BDNF-induced spikes during current injection was significantly higher in inflamed rats. The BDNF-induced changes in TRG neuron excitability was abolished by tyrosine kinase inhibitor, K252a.

**Conclusion:**

The present study provided evidence that BDNF enhances the excitability of the small-diameter TRG neurons projecting onto the Vi/Vc following MM inflammation. These findings suggest that ganglionic BDNF-trkB signaling is a therapeutic target for the treatment of trigeminal inflammatory hyperalgesia.

## Background

Although it is well known that the spinal trigeminal nucleus caudalis (SpVc) and the upper cervical spinal dorsal horn (C1-2) are important relay stations for trigeminal nociceptive inputs from inflammation and injury in superficial and deep tissues [1,2], it has also been reported that the nociceptive inputs from receptors in deep craniofacial tissues are relayed to the ventral trigeminal subnucleus interpolaris/caudalis transition region (Vi/Vc) through the trigeminal subnucleus caudalis/cervical dorsal horn C_2_ (Vc/C_2_) junction region [3]. Recent studies demonstrate that orofacial injury and noxious stimulation of dental and craniofacial region activates a distinct region in the Vi/Vc transition zones [4-6]. Indeed, the injection of an N-methyl-D-aspartate (NMDA) receptor antagonist into the Vi/Vc transition zone attenuates hyperalgesia after masseter muscle (MM) inflammation [7]. Sugiyo et al. [5] demonstrated that reciprocal connections between the ventral Vi/Vc transition zone and rostral ventromedial medulla (RVM) pathways play a critical role in facilitating orofacial hyperalgesia. These findings suggest that the trigeminal Vi/Vc transition zone plays an important role in deep tissue pain processing, integrating nociceptive orofacial pain inputs and the development of persisting orofacial pain [6].

Since previous studies have demonstrated that non-synaptically-released diffusible chemical messengers, such as adenosine triphosphate (ATP), substance P (SP) and glial-derived neurotrophic factor (GDNF), act via local paracrine mechanisms in the sensory ganglia to contribute to the development of inflammation-induced sensory abnormalities [8-12], it can be assumed that transganglionic communication is one mechanism by which central sensitization can be triggered, and that suppression of ganglionic sensitization can effectively reduce the central sensitization of secondary neurons [13,14]. Brain-derived neurotrophic factor (BDNF) facilitates pain transmission and contributes to the development of hyperalgesia [15-18] via the postsynaptic tyrosine kinase B (trkB) receptor to modulate nociceptive signaling in the spinal dorsal horn. BDNF is normally expressed in small- and medium-sized dorsal root ganglion (DRG) and trigeminal ganglion (TRG) neurons [19-21], and is localized to dense-core vesicles in axon terminals in the SpVc region [22]. Recent studies report upregulation of BDNF and trkB immunoreactivity (IR) in TRG neurons after tooth pulp inflammation or injury [23,24]. These findings suggest that local release of BDNF from TRG neuronal somata and/or nerve terminals may regulate the excitability of TRG neurons projecting onto the Vi/Vc transition zone following deep tissue inflammation and may contribute to the development of hyperalgesia. Therefore, the present study investigated the functional significance of the BDNF-trkB signaling system in TRG neurons projecting to the Vi/Vc transition zone on trigeminal hyperalgesia following MM inflammation.

## Methods

All experiments were approved by the Animal Use and Care Committee of Nippon Dental University and were consistent with the ethical guidelines of the International Association of the Study of Pain [25]. Each experiment was performed such that the experimenter was blind to experimental conditions. Every effort was made to minimize the number of animals used and their suffering.

### Induction of masseter muscle inflammation

The experiments were performed on 18 adult male Wistar rats (110-150 g; naïve, n = 8; inflamed, n = 10). Each animal was anesthetized with sodium pentobarbital (45 mg/kg, i.p.), and complete Freund’s adjuvant (CFA) (0.05 ml 1:1 oil/saline suspension; inflamed rats) or vehicle (0.05 ml, 0.9% NaCl, pH 7.2; naïve rats) was injected into the left side of the MM, as described previously [26]. In some experiments (n = 2), the CFA-induced inflammation was verified with Evan’s blue dye (50 mg/ml, 1 ml/kg, i.v.) extravasation. Postmortem examination of the injected MM showed the accumulation of blue dye in the MM, indicating that the plasma protein extravasation was due to localized inflammation [10].

### Double labeling of TRG neurons innervating MM and/or projecting to the Vi/Vc region

For electrophysiological and immunohistochemical studies, fluorogold (FG, Fluorochrome, Englewood, USA) and fluorescent latex microbead (MB, Lumafluor, Naples, FL) labeling methods [27] were used. Male Wistar rats were anaesthetized with pentobarbital sodium (45 mg/kg, i.p.) before FG solution (0.5% in distilled water, 10 μl) was injected into the left MM using a Hamilton syringe with a 31-gauge needle. The dorsal surface of the medulla oblongata at the obex level was then surgically exposed and MB (0.05 μl) was injected ipsilaterally into the Vi/Vc region (obex +0.5 mm, lateral 0.5 mm, depth 0.5 mm) by pressure injection through a glass micropipette (tip diameter of 30–50 μm) as described previously [5,27-29]. After MB injection, the muscle and skin incisions were sutured and the rats were allowed to recover. The injection sites and spread of MB were verified by histology.

### Mechanical threshold for escape behavior

The mechanical threshold for escape behavior was determined as described in previous studies [12]. Briefly, two days after the injection of CFA or vehicle into the MM, hyperalgesia was assessed with calibrated von Frey filaments (Semmes-Weinstein Monofilaments, North Coast Medical, CA). To evaluate the escape threshold, a set of the von Frey mechanical stimuli were applied to the skin overlying the MM in an ascending series of trials. Each von Frey stimulation was applied three times in each series of trials. The escape threshold intensity was determined when the rat moved its head away from at least one of three stimuli.

### BDNF and trkB immunohistochemistry

Immunohistochemistry was used to determine whether inflammation induces BDNF and trkB expression in the trigeminal ganglia in both naïve (n = 3) and inflamed (n = 3) rats. Two days after CFA or vehicle injection rats were anesthetized (sodium pentobarbital; 50 mg/kg, intraperitoneal), and transcardially-perfused with 1% sodium nitrite in phosphate buffered saline (PBS; 50 ml) followed by Zamboni fixative (4% paraformaldehyde, 15% picric acid in 0.1 M phosphate buffer). Tissue was post-fixed for up to 2 hours. The left TRG was removed and incubated, in sequence, in 4% (5 min × 3), 10% (1 h), 20% (2 h) and 30% (overnight) sucrose solution. Sections were cut (10 μm) with a cryostat (Leica, Nussloch, Germany) and mounted on silane-coated glass slides.

Every third section was used for immunohistochemistry using a modification of the method previously described [10], with twenty sections analyzed per ganglion. Briefly, sections were incubated with sheep anti-BDNF(1:200, Millipore, CA, USA) and rabbit anti-trkB (1:1000, Millipore, CA, USA) for 24 h at 4°C, followed by Alexa Fluor® 568 donkey anti-sheep IgG (1:1000, Molecular Probes, Eugene, OR, USA) and Alexa Fluor® 647 goat anti-rabbit IgG (1:1000, Molecular Probes, Eugene, OR, USA), respectively. Labeled cryosections were rinsed in 0.01 M PBS, 5 min, and mounted with antifade medium (Molecular Probes). Control sections were incubated without the primary antibody.

The number of BDNF/trkB-IR TRG neurons was counted in each section. Immunopositive staining was compared with background staining by comparing least intense staining to that with primary antibody omitted [10]. In this study, we determine the fluorescence wave lengths of both BDNF and trkB to prevent interference. Digital images were collected and stored on a computer and analyzed with Adobe Photoshop and a Leica Imaging Analysis Tool. Confocal images were generated with a Leica TCS NT laser scanning microscope (Leica, Germany).

### Acute cell dissociation and whole cell patch-clamp recording for TRG neurons

Patch-clamp recordings were conducted two days after CFA or vehicle injection, on naïve (n = 5) and inflamed rats (n = 7). Acute dissociation of TRG neurons was performed as described previously [10]. Briefly, adult rats were decapitated and the left TRG rapidly removed and incubated for 15–25 min at 37°C in modified Hank’s balanced salt solution (130 mM NaCl, 5 mM KCl, 0.3 mM KH_2_PO_4_, 4 mM NaHCO_3_, 0.3 mM Na_2_HPO_4_, 5.6 mM glucose, 10 mM N-2-hydroxyethylpiperazine-N’-2-ethanesulfonic acid (HEPES), pH 7.3) containing type XI collagenase (2 mg/ml; Sigma, St. Louis MO, USA) and type II collagenase (2 mg/ml; Sigma). Cells were dissociated by trituration with a fire-polished Pasteur pipette and plated onto poly-L-lysine-coated coverslips in 35 mm dishes. The plating medium contained Leibovitz’s L-15 solution (Invitrogen, Carlsbad, CA, USA) supplemented with 10% newborn calf serum, 26 mM NaHCO_3_ and 30 mM glucose. Cells were maintained in 5% CO_2_, 37°C and used for recording 2-8 h after plating. After incubation, the coverslips were transferred to the recording chamber in a standard external solution containing 155 mM NaCl, 3 mM KCl, 1 mM CaCl_2_, 1 mM MgCl_2_, 10 mM HEPES and 20 mM glucose, pH 7.3. The recording chamber (0.5 ml) was mounted onto an inverted microscope (Nikon, Tokyo, Japan) equipped with phase contrast, a video camera and two micromanipulators. The chamber was perfused under gravity with a standard external solution at approximately 0.5 ml/min.

Whole cell recordings were conducted with the rapid perforated-patch technique [10,27]. Fire-polished patch-pipettes (2–5 MΩ) were filled with 120 mM potassium methanesulphonate, 20 mM KCl, 7.5 mM HEPES and 2 mM EGTA (ethylene glycol-bis-β-aminoethyl ether N,N,N’,N’-tetra-acetic acid), pH 7.3, containing amphotericin B (100 μg/ml). Current-clamp recordings were conducted with an Axopatch 200B amplifier (Molecular Devices, Foster City, CA, USA). Signals were low-pass filtered at 1 or 5 kHz and digitized at 10 kHz. To evaluate changes in cell membrane resistance associated with membrane potential during recordings in the current-clamp mode, negative current pulses (50–600 pA, 250 ms, 0.2 Hz) were injected through the patch pipette. Action potentials (overshoot of action potential > 0 mV) were elicited by depolarizing current pulses (10–500 pA, 10-70 pA steps, 200 ms). The threshold for action potentials was determined and defined as the minimum current required for eliciting a single spike. The tetrodotoxin (TTX) 1 μM sensitivity for TRG neurons was tested, with neurons defined as TTX-resistant (TTX-R) if no changes of spike amplitude elicited by threshold depolarizing current pulses were found in the presence of TTX [30]. The action potential firing rates were assessed by counting the number of action potentials elicited by depolarizing pulses (2-times threshold currents). Spike duration was determined as the duration of the first spike at the level of half amplitude. Access resistance was checked throughout the experiments, and no significant changes found during experiments. All recordings were performed at room temperature. Since spontaneous fluctuations in the membrane potential were less than 1 mV, a depolarizing response is defined as over 1 mV. The threshold for BDNF response (changes in the membrane potential) of each neurons tested was determined by minimum concentration of BDNF (1–100 ng/ml). After confirmation of a BDNF responsive cell, the tyrosine kinase inhibitor K252a was applied. In the current-clamp mode, the membrane potential in most cells recovered within 5–14 min.

### Drug application

All drugs were stored at −20°C and stock solutions dissolved in distilled water. Drugs were subsequently dissolved in a standard external solution on the day of the experiment. BDNF (1-100 ng/ml; Sigma-Aldrich, St. Louis, MO, USA) and tyrosine kinase inhibitor K252a (10 ng/ml; Sigma-Aldrich) were added to the perfusion solution for a period of 30–60 sec.

### Data analysis

Data were stored on a computer for off-line analysis (pClamp 8.0, Molecular Devices). Values are expressed as mean ± SEM. Statistical analyses were performed using Student’s te-test and one-way repeated measures analysis of variance (ANOVA) followed by the Tukey-Kramer/Dunnett’s post hoc tests for the data. *P* < 0.05 was considered statistically significant.

## Results

### Induction of MM inflammation and mechanical hyperalgesia

**Figure 1:**
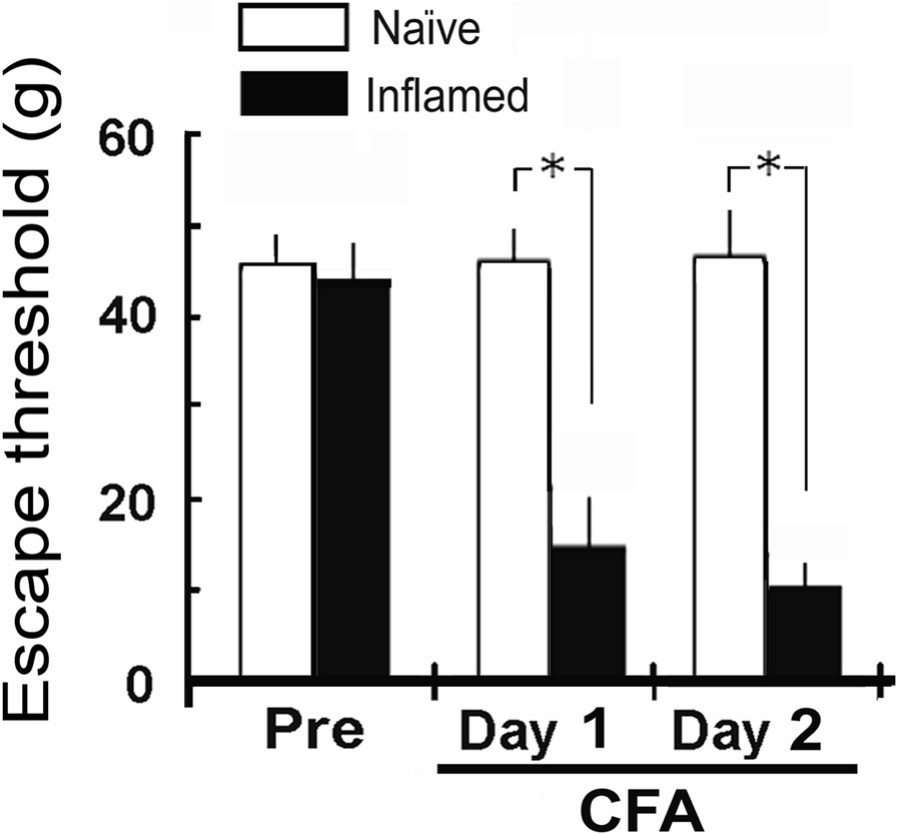
**Changes in the mechanical escape threshold after induction of MM inflammation and hyperalgesia.** Two days after complete Freund’s adjuvant (CFA) or vehicle was injected into the skin overlying the ipsilateral MM, mechanical stimulation using von Frey filaments was applied to assess hyperalgesia in naïve and inflamed rats. Data are the mean ± SEM (naïve, *n* = 8; inflamed, n = 10). *P < 0.05.

After CFA injection into the MM region, animals showed behavioral hyperalgesia, determined by probing the injected site (the left side) overlying orofacial skin with von Frey filaments. In inflamed rats, the threshold for escape from mechanical stimulation applied to the skin above the MM was significantly reduced one and two days after CFA injection (2 days; 46.9 ± 8.2 to 9.5 ± 3.5 g, *P* < 0.05; Figure 1).

### Retrograde labeling of TRG neurons innervating MM and/or projecting to the Vi/Vc region

**Figure 2:**
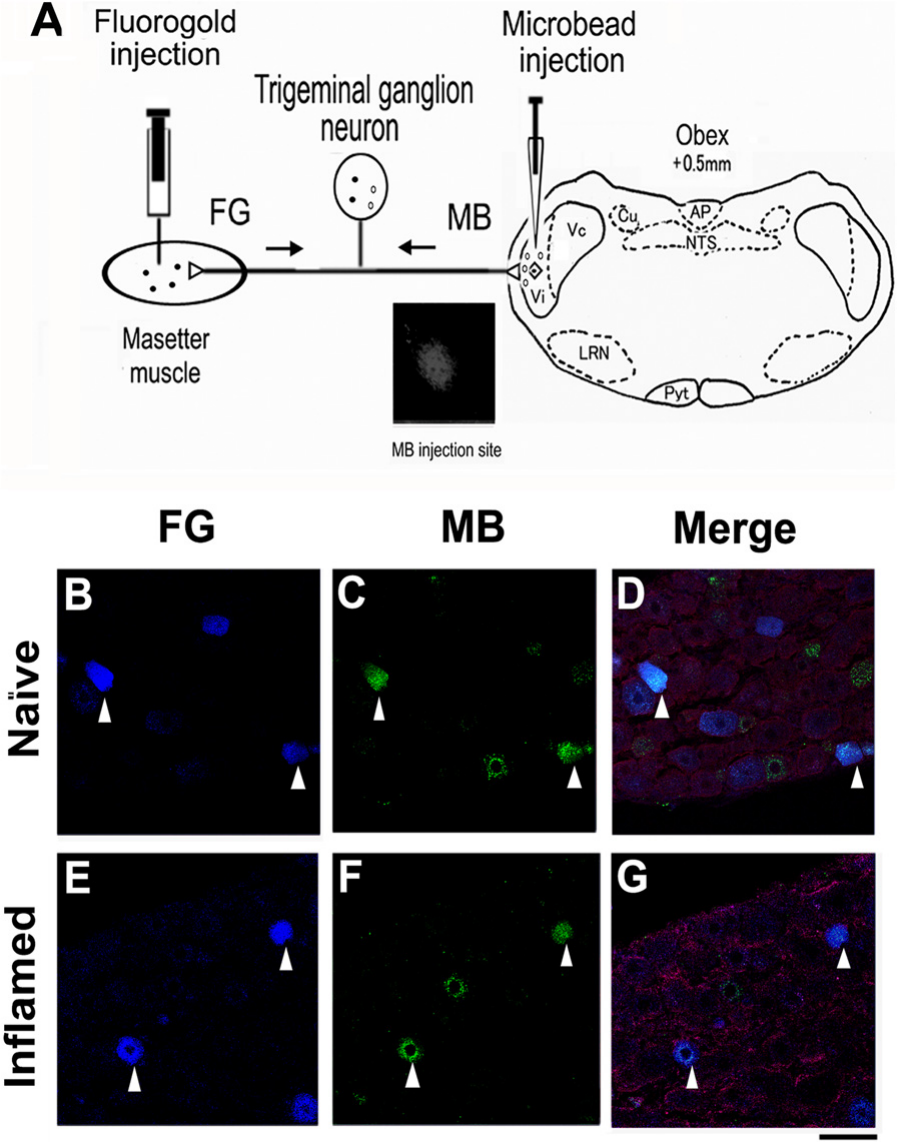
**Schematic drawing of double labeling of TRG neurons and an example of FG/MB-labeled TRG neurons. *A***: TRG neurons innervating the masetter muscle (MM) were labeled by fluorogold (FG) and the same neurons projecting to the trigeminal subnucleus interpolaris/caudalis transition region (Vi/Vc) were labeled by fluorescent latex microbeads (MB). Inset show the location of MB injection sites. AP, area postrema; NTS nucleus of tractus solitaries; Cu, cunate nucleus, LRN, Lateral reticular nucleus; Pyt, pyramidal tract. **B**-**C***:* TRG neurons that innervated masseter muscle (MM) **(B)** projected onto the Vi/Vc region **(C)** in naïve rats. **E**-**F**: TRG neurons that innervated masseter muscle (MM) **(E)** projected onto the Vi/Vc region **(F)** in inflamed rats**. *B***,***E****:* Fluorescence micrograph showing FG-labeled TRG neurons. ***C***,***F****:* MB-labeled TRG neurons in the same section. ***D ***and ***G***: Merge. Triangle shows an example of FG/MB-labeled TRG neurons. Bar = 20 μm.

Based on our previous immunohistochemical examinations [44], TRG cell bodies were classified according to size as small (<30 μm), medium (30–39 μm) or large (>40 μm) in this study. Figure 2A shows schematic drawing of double labeling of TRG neurons and an example of FG/MB-labeled TRG neurons. In naïve rats, two days after the FG injection into the MM, 19.5% of TRG neurons (751/3850) innervating MM were retrogradely labeled by FG (Figures 2B-D), similarly to described in our previous studies [27]. Approximately half of these neurons were also labeled with the MB (44.1%, 331/751) and projected onto the Vi/Vc region (Figures 2C, 2D), similarly to as described previously [7]. These neurons ranged from small to medium in diameter (<39 μm). Similarly, in inflamed rats, 20.0% of TRG neurons innervating MM were retrogradely labeled by FG (733/3669; Figure 2E). Approximately half of these neurons ranged from small to medium in diameter, were labeled with the MB retrograde tracer (47.9%, 351/733), and projected onto the Vi/Vc region (Figure 2F, 2G).

### Inflammation increases BDNF expression in FG/MB labeled TRG neurons

**Figure 3:**
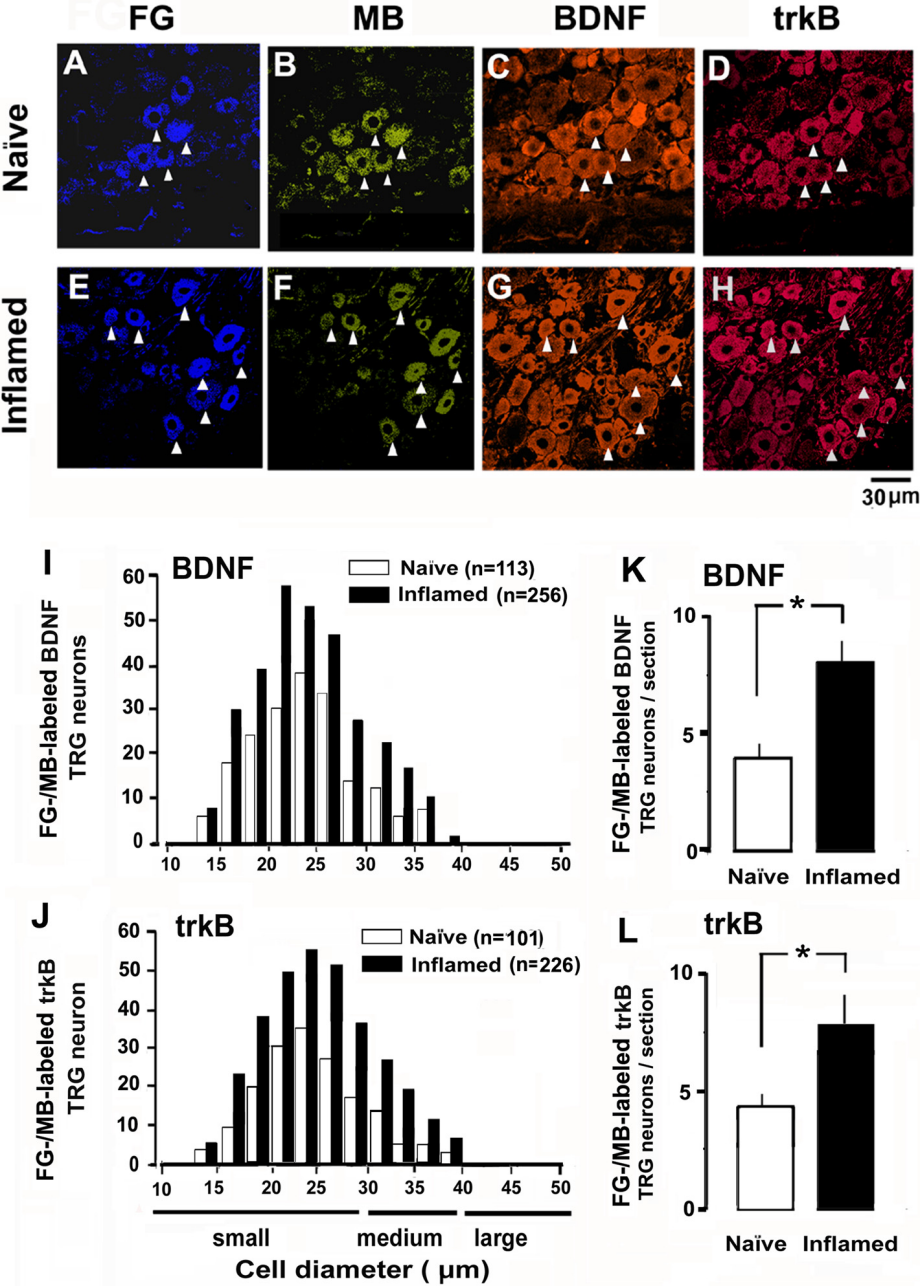
**Difference between expression of BDNF and trkB in TRG neurons in naïve and inflamed rats. *A***-***D****:* Naïve rats. FG/MB-labeled TRG neurons innervating MM **(*A*)** and projecting to Vi/Vc **(*B*)**. Triangle shows an example of FG/MB-labeled and BDNF/trkB-IR TRG neurons. ***E***-***H****:* Inflamed rats. FG/MB-labeled TRG neurons innervating MM **(C,D) (*E*)** and projecting to Vi/Vc **(*F*)**. Triangle shows an example of FG/MB-labeled and BDNF/trkB-IR TRG neurons. Bar = 20 μm. ***I****:* Size frequency distribution of FG/MB-labeled BDNF-IR TRG neurons in naïve and inflamed rats ***(G,H)***. ***K***: Comparison of FG/MB-labeled BDNF-IR TRG neurons per section in both naïve and inflamed rats. *P < 0.05. ***J****:* Size frequency distribution of FG/MB-labeled trkB-IR TRG neurons in naïve and inflamed rats. ***L***: Comparison of FG/MB-labeled trkB-IR TRG neurons per section in both naïve and inflamed rats.*P < 0.05.

In both naïve and inflamed rats, the location of FG/MB-labeled BDNF-IR neuronal soma corresponded with those in the third branch of the trigeminal nerve [10,31]. Figures 3A-G shows a typical example of FG/MB labeled BDNF-IR TRG neurons in naïve and inflamed rats. The FG/MB-labeled and BDNF-IR TRG neurons were small-medium in diameters in naïve rats (34.1%, 113/331) as described previously [21]. The size frequency of distribution of FG-/MB-labeled/BDNF-IR TRG neurons is shown in Figure 3I. The number of small-medium diameter BDNF-expressing neurons in the FG/MB-labeled TRG increased in the inflamed rat (72.9%: 256/351) compared to the naïve rat. The number of FG/MB-labeled BDNF-IR neurons was significantly higher in inflamed rats than naïve (naïve vs inflamed: 4.2 ± 0.9/section vs 8.5 ± 1.1/section, *P* < 0.01; Figure 3K). In the absence of primary antibody only background staining was evident (data not shown).

### Inflammation increases trkB expression in FG/MB-labeled TRG neurons

Similarly to the BDNF-IR TRG neurons, the location of FG/MB-labeled trkB-IR neuronal soma corresponded with those in the third branch of the trigeminal nerve. Examples of FG-/MB-labeled trkB-IR TRG neurons in naïve and inflamed rats are shown in Figures 3A, 3B, 3D and Figures 3E, 3F, 3H, respectively. The FG/MB-labeled and trkB-IR TRG neurons were small-medium in diameters in naïve rats (31%, 101/331). Importantly, the majority of FG/MB-labeled BDNF-IR neurons also expressed trkB (naïve 86%; inflamed 93%). The size frequency of distribution of FG-/MB-labeled/trkB-IR TRG neurons is shown in Figure 3J. The number of small-medium diameter trkB-expressing neurons in the FG-/MB-labeled TRG increased in the inflamed rat (66%: 226/351) compared to the naïve rat. The number of FG/MB-labeled trkB-IR neurons was significantly higher in inflamed rats than naïve rats (naïve vs inflamed: 4.6 ± 0.5/section vs 8.9 ± 1.3/section, *P* < 0.01; Figure 3L). In the absence of the primary antibody only background staining was evident (data not shown).

### General electrophysiological properties of FG/MB-labeled TRG neurons

**Figure 4:**
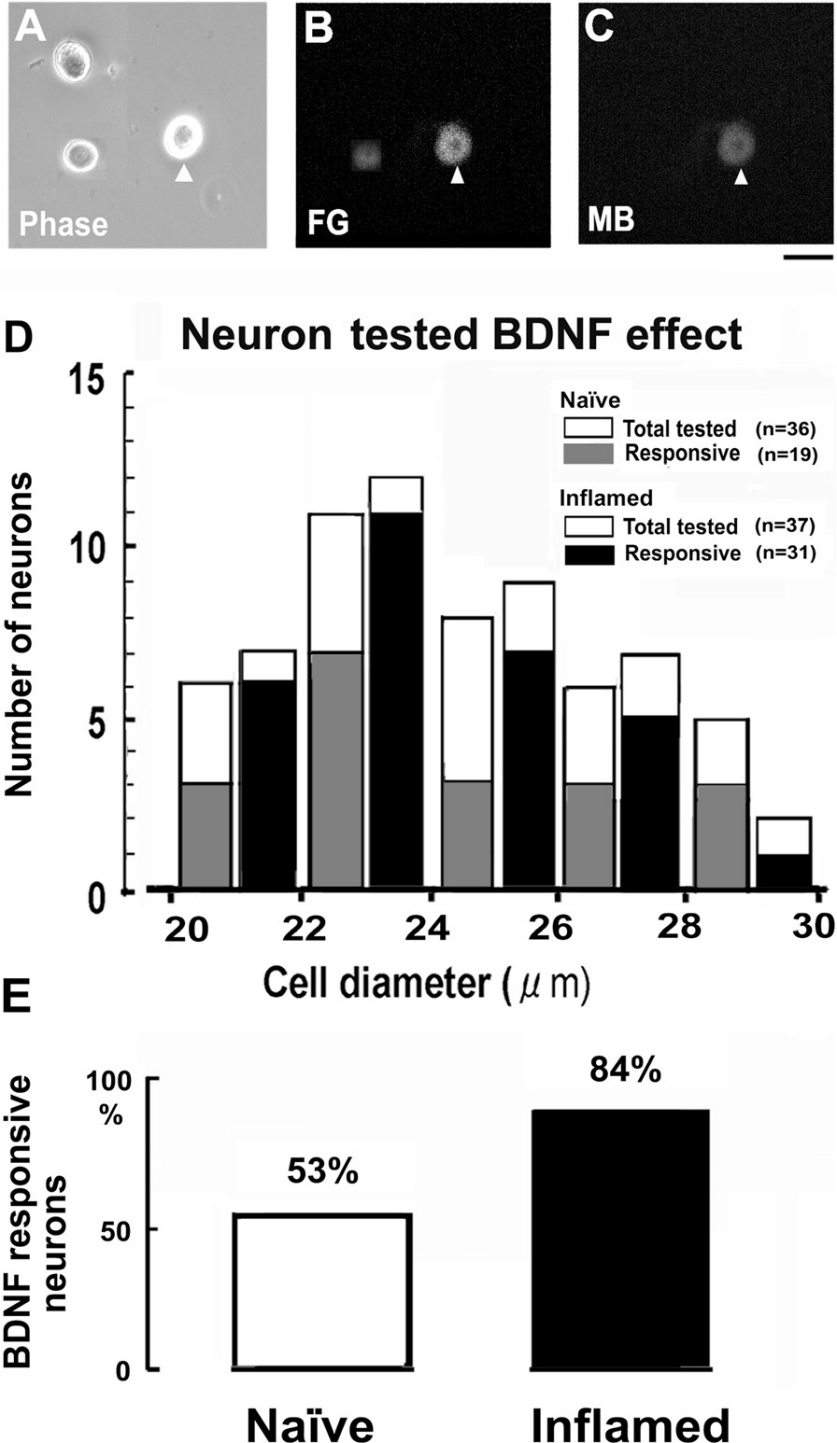
**Double-labeling of dissociated TRG neurons recorded under current-clamp and BDNF responsiveness in naïve and inflamed rats.** Acutely dissociated TRG neurons observed under phase contrast microscopy **(*A*)**, identified by FG-injection in the MM **(*B*)** and by MB injection into the Vi/Vc region in the same field ***(C)***. Bar = 20 μm. Arrow heads denote the target cell following FG/MB injection. ***D****:* Size distribution of dissociated FG/MB-labeled small-diameter TRG neurons recorded in both naïve and inflamed rats. BDNF responsiveness was determined by changes in the membrane potentials (>1 mV). ***E****:* Percentage of BDNF responsiveness in FG/MB-labeled small-diameter TRG neurons.

Based on the upregulation of BDNF and trkB expression in small-medium diameter TRG neurons after inflammation, and our interest in inflammatory hyperalgesia, we used small-diameter TRG neurons for the electrophysiological recordings. The isolated FG/MB-labeled TRG neurons were spherical in shape and bright in appearance with a 'halo’ around the cell body when viewed by phase contrast microscopy (Figures 4A-C). Figure 4D shows the size distribution of recorded FG/MB-labeled TRG neurons responding to 50 ng/ml BDNF (responding cell; naïve 19/36, 53%; inflamed 31/37, 84%). Since previous studies have suggested that BDNF may cause significant changes in neuronal excitability of DRG neurons [32] and Purkinje cells in the cerebellum [33], we used this concentration to test BDNF responsiveness of TRG neurons. The mean cell diameter of FG/MB-labeled TRG neurons used for electrophysiological recordings in naïve and inflamed rats was 26.5 ± 4.8 μm (*n* = 73). There was a tendency for the BDNF responsiveness to be greater in inflamed rats than in naïve rats. Following perforation of the cell membrane with amphotericin B in dissociated TRG neurons (naïve, n = 36; inflamed, n = 37), the series resistance dropped to below 20 MΩ (17.1 ± 5.3 MΩ, *n* = 73) within 3–9 min and remained stable for over 15 min. The mean cell capacitance was 15.2 ± 4.9 pF (*n* = 73).

### Effect of BDNF on the resting membrane potential of small-diameter FG/MB-labeled TRG neurons in naïve and inflamed rats

To determine whether BDNF alters the excitability of TRG neurons, the effect of BDNF on the resting membrane potential of FG/MB-labeled small-diameter TRG neurons in naïve and inflamed rats was examined. Since BDNF depolarized the majority of neurons (naïve 14/19, 74%; inflamed 26/31, 84%) and the remaining neurons were hyperpolarized after BDNF application (naïve 5/19, 27%; inflamed 5/31, 13%), in this study we focused on the depolarization response.

**Figure 5:**
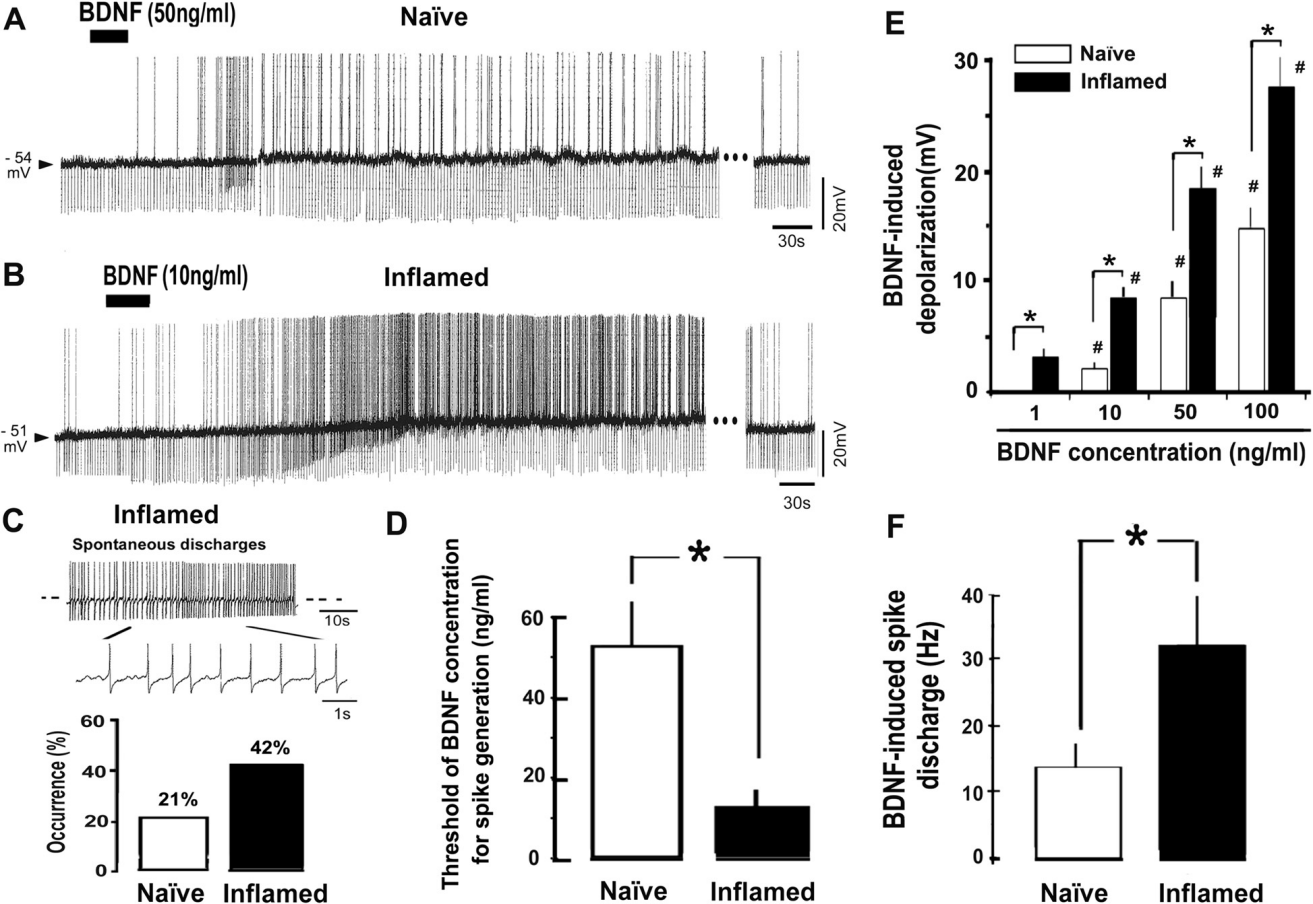
**Effect of BDNF on the neuronal excitability of small-diameter FG/MB-labeled TRG neurons in naïve and inflamed rats. *A****:* BDNF (50 ng/ml) induced neuronal depolarization with spike discharges in FG/MB labeled TRG neurons in naïve rats. The break in the trace is 5 min. ***B****:* BDNF(10 ng/ml) induced strong neuronal depolarization with spike discharges in small-diameter FG/MB labeled TRG neurons in inflamed rats. The break in the trace is 13 min. ***C****:* Spontaneous discharge in small-diameter FG/MB labeled TRG neurons in inflamed rats. The percentage and mean firing frequency of TRG exhibiting spontaneous discharges increased following inflammation. The break in the trace is 7 min. ***D****:* The threshold concentration of BDNF required for spike generation depolarization in inflamed rats was significantly lower than in naïve rats. ***E****:* Dose–response relationship for BDNF-induced depolarization in naïve and inflamed rats. (n = 4, #, p < 0.05, 1 vs 10, 50 and 100 ng/ml, *, p < 0.05, naïve vs inflamed). ***F****:* Comparison of discharge frequency induced by BDNF (50 ng/ml) in naïve and inflamed rats. *P < 0.05.

Figure 5A shows a typical example of the depolarizing response of FG/MB-labeled TRG neurons to BDNF (50 ng/ml) with associated decreasing input resistance, in naïve rats. The duration of the depolarizing response ranged from 4 to 8 min. Figure 5B shows a typical example of a strong depolarization response to BDNF (10 ng/ml) which induced increments in spike discharge frequency and was associated with a decrease in input resistance in inflamed rat TRG neurons. The duration of the BDNF-induced depolarizing response in inflamed rats was significantly longer than in naïve rats (naïve vs inflamed; 4.8 ± 2.2 min vs 8.6 ± 3.1 min, *P* < 0.05). Although relatively few neurons (3/14, 21%) in naïve rats showed spontaneous discharges, there was a two-fold increase in the number of TRG neurons (11/36, 42%) having spontaneous discharges (Figure 5C) in the inflamed rats, and the discharge rate was greater than in naïve rats (naïve vs inflamed; 0.8 ± 0.4 Hz, n = 3, vs 2.6 ± 1.1 Hz, n = 11; *P* < 0.05), supporting previous findings [11]. The threshold concentration of BDNF that evoked a depolarizing response in the inflamed rats was significantly lower than in naïve (naïve vs inflamed; 52.3 ± 14 ng/ml vs 12.9 ± 6.3 ng/ml; *P* < 0.05; Figure 5D). This depolarizing response was associated with a decrease in cell input resistance and this decreasing input resistance was significantly higher in inflamed rats than in naïve rats (naïve vs inflamed; 11.4 ± 2.3% vs 25.2 ± 5.5%; *P* < 0.05). BDNF-induced depolarization was significantly increased in dose-dependent manner in both naïve and inflamed rats (1–100 ng/ml) and the mean depolarization of FG/MB-labeled TRG neurons induced by BDNF in inflamed rats was significantly higher than in naïve rats (Figure 5E). The mean firing frequency of inflamed rat FG/MB-labeled TRG neurons evoked by BDNF was significantly higher than in naïve rats (Figure 5F). The mean neuronal resting membrane potential in inflamed rats was not significantly different to naïve rats (naïve vs inflamed; -55.2 ± 2.9 mV, n = 16 vs −52.1 ± 2.3 mV, *n* =26; NS; Figure 5F), as described in a previous study [30].

### Effect of BDNF on the firing rate of small-diameter FG/MB-labeled TRG neurons in naïve and inflamed rats

**Figure 6:**
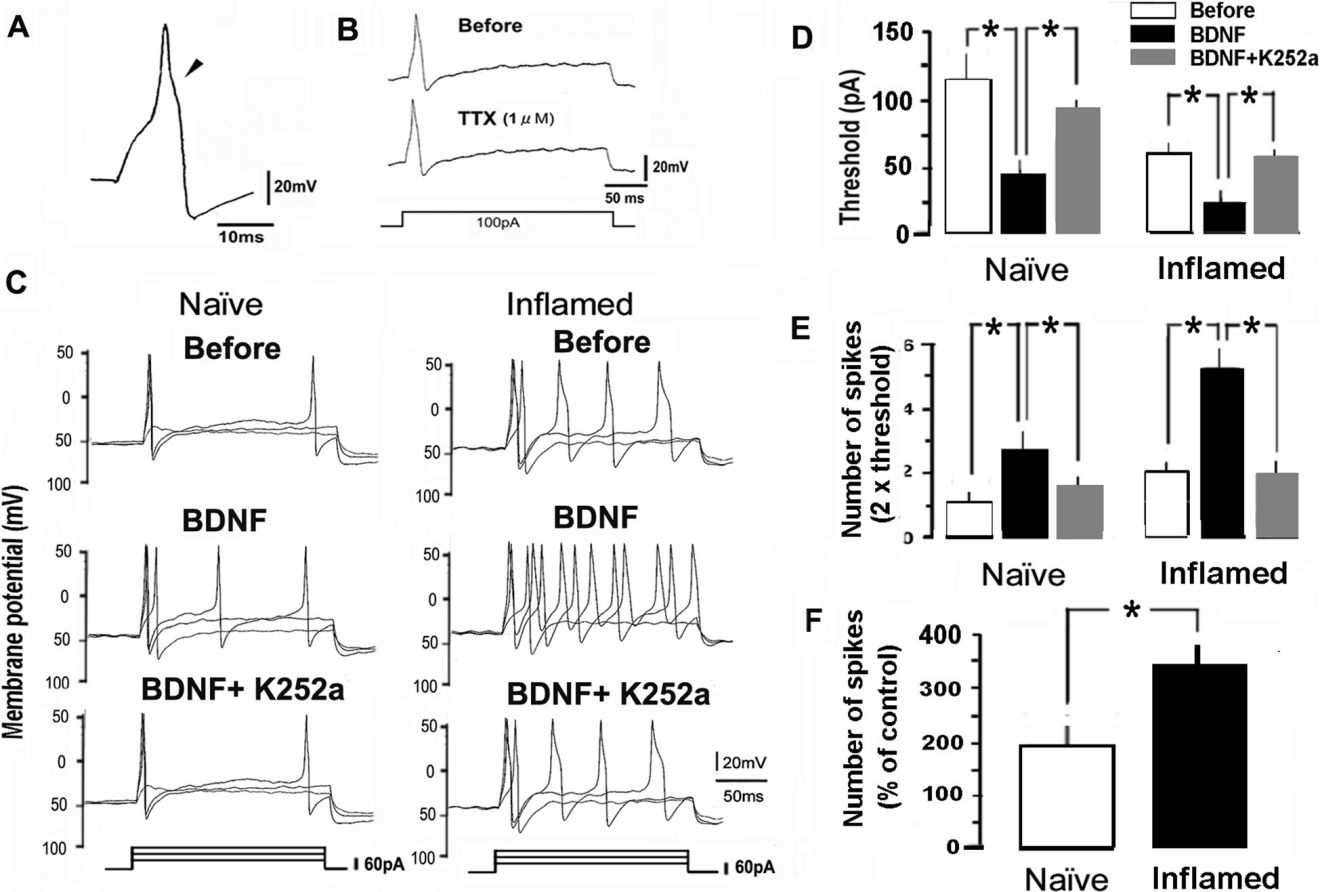
**Comparison of effect of BDNF on the firing frequency of small-diameter FG/MB-labeled TRG neurons from naïve and inflamed rats. *A****:* Wave form of action potential in response to depolarizing current pulses. Note the prominent shoulder on the falling phase (triangle). ***B****:* Action potential induced at threshold level (300 ms, 100 pA) which was resistant to TTX (1 μM). ***C****:* Action potential firing rate during depolarization of step pulse before (upper trace) and after (middle trace) BDNF (50 ng/ml), and after BDNF (50 ng/ml) + K252a (10 ng/ml; lower trace) application to small-medium diameter TRG neurons in naïve and inflamed rats. ***D****:* Threshold current for spike generation during current injection before and after BDNF and after BDNF + K252a applications; *P* < 0.05. ***E****:* Number of spike discharges during current injection before and after BDNF and after BDNF + K252a applications; *P* < 0.05. ***F****:* The relative number of spikes during current injection (100 pA, 200 ms) was significantly higher in inflamed rats than in naïve rats. *P < 0.05.

To determine whether MM inflammation alters spike generation evoked by BDNF in FG/MB-labeled small-diameter TRG neurons, the changes in action potential firing rate induced by a depolarizing step pulse was examined in the absence and presence of BDNF in naïve and inflamed rats. Small-diameter FG/MB-labeled TRG neurons had several characteristics typical of nociceptors: (1) small size (<30 μm); (2) presence of long-duration action potentials [34]; and (3) resistance of the action potential to TTX [35,36]. A typical example of the wave form of an action potential is shown in Figure 6A. In current-clamp mode, FG/MB-labeled small-diameter TRG neurons exhibited a long duration (4.3-8.8 ms) with a prominent shoulder on the repolarization phase (naïve, n = 7; inflamed, n = 10). All neurons tested exhibited action potentials in the presence of TTX (1 M; Figure 6B).

Figure 6C shows a typical example of the effect of BDNF and co-application of K252a on the spike generation during depolarizing pulses in small-diameter FG/MB-labeled TRG neurons in naïve and inflamed rats. Similarly to a previous study [30], in the present study we observed; (1) the threshold current of FG/MB-labeled TRG neurons in inflamed rats was significantly decreased compared to naïve rats (121.2 ± 12.4 pA vs 61.1 ± 8.9 pA; *P* < 0.05; Figure 6C, upper trace and Figure 6D); (2) the number of spike discharges during current injections by FG/MB-labeled TRG neurons in inflamed rats was significantly increased compared to naïve rats (1.2 ± 0.3 spikes vs 2.1 ± 0.2 spikes; *P* < 0.05; Figure 6C, upper trace and Figure 6E). In both naïve and inflamed rats, the number of spike discharges during the current injection significantly increased after BDNF (50 ng/ml) application (*n* = 5, *P* < 0.05; Figure 6C middle trace and Figure 6E). The threshold current of spike generation significantly decreased after BDNF application in both naïve and inflamed rats (*n* = 5, *P* < 0.05; Figure 6D). The relative number of spikes during current injection (100 pA, 200 ms) was significantly higher in inflamed rats than in naïve rats (355.2 ± 41.4% vs 205.3 ± 38.9%, respectively, *n* = 5, *P* < 0.05; Figure 6F). The decreased threshold current evoked by BDNF was blocked by co-administration of the tyrosine kinase inhibitor, K252a (10 ng/ml) in both naïve and inflamed rats (Figure 6C*,* lower trace and Figure 6D). Increased spike discharges evoked by BDNF were also blocked by co-administration of K252a (10 ng/ml) in both naïve and inflamed rats (Figure 6C*,* lower trace and Figure 6E).

## Discussion

### Inflammation upregulates BDNF/trkB expression in TRG neurons innervating MM and projecting to the Vi/Vc

By means of double-labeling tracing techniques with FG and MB, we observed that approximately half of TRG neurons identified masseteric input to ventral Vi/Vc transition zone in this study. This was consistent with the report that both masseter and cutaneous inputs to project the Vc, masseter afferents provide an addtional input to the Vi/Vc [37]. Since the location of deposits of MB was localized in the Vi/Vc region (Figure 2), it is unlikely that MB was taken up by damaged fibers that course laterally through the region and that the tracer spreads to the spinal trigeminal tract. Also, there was no significant difference in the percentage of FG/MB-labeled neurons between naïve and inflamed rats (*P >*0.05). Taken together, therefore, our double-retrograde labeling methods are a valid technique for examining the primary afferent TRG neurons innervating MM and projecting to the Vi/Vc region.

BDNF is expressed in small-medium diameter DRG neurons that also express tyrosine kinase A (trkA) and calcitonin gene related peptide (CGRP). BDNF synthesized in the DRG [19,38,39] was transported to the central terminals of primary afferents [19,38,39] and released into the spinal dorsal horn, where it bound to the trkB receptors on the second order neurons to modulate painful stimuli [40-42]. Similarly, small-medium diameter TRG neurons also expressed both BDNF and trkB in naïve rats [21,43,44]. Furthermore, we found that most of FG/MB-labeled small-medium diameter TRG neurons co-expressed BDNF and trkB and that this expression was predominantly co-localised (naïve 86%; inflamed 93%).

BDNF and trkB mRNA levels are reported to be significantly increased in DRG neurons in a CFA inflammation model [41,45,46]. BDNF and trkB in small-medium diameter TRG neurons is known to be upregulated after inflammation and tooth-injury, and BDNF is released from TRG neurons in response to neuronal hyperactivity [22-24]. The inflammation-induced upregulation of BDNF is hypothesized to be mediated by NGF and hyperalgesia [41]. Peripheral inflammation and injury are also associated with the release of pro-inflammatory cytokines such as tumor necrosis factorα (TNFα) and interleukin 1β (IL-1β) [47]. Recently, Balkowiec-Iskra et al. [48] reported that TNFα upregulated BDNF expression in TRG neurons via p38-mitogen-activated protein kinase (MAP) in an 'NGF-independent manner’, leading to trigeminal hyperalgesia. This study shows that two days after CFA-induced MM inflammation, the mean number of BDNF- and trkB-IR small-medium diameter TRG neurons in inflamed rats was significantly higher than in naïve rats. Therefore, taken together, these observations suggest that both NGF and cytokine signaling may upregulate BDNF and trkB in small-medium diameter TRG neurons to induce hyperalgesia. This is also supported by a recent report that, under inflammatory conditions, enhanced production of BDNF in DRG neurons might activate presynaptic trkB receptors via an autocrine/paracrine signal to synergistically act with cytokines such as TNFα, and upregulate synaptic excitability in pain transmission [49].

### Changes in the excitability of TRG neurons via upregulation of BDNF and trkB

Although we have no evidence that release of BDNF from TRG neurons into the TRGs is increased following peripheral inflammation in this study, there are reports that primary sensory neurons can release endogenous BDNF in activity-dependent manner, and that the magnitude of release dependents on the pattern and frequency of stimulation [50,51]. Thus, it can be assumed that TRG neurons contain BDNF secrete BDNF into the extracellular space in the TRGs following neuronal excitation, resulting in an increase in BDNF concentration in the extracellular space.

Previous studies have indicated that BDNF is not only a potent neuromodulator, but also has a fast excitatory action on neurons which acutely controls resting membrane potential, neuronal excitability and synaptic transmission, and also participates in the induction of synaptic plasticity [52]. With regards to the effect of BDNF on nociceptive transmission, Matayoshi et al. [16] reported that in spinal cord slice preparations, BDNF acted on presynaptic terminals and increased the frequency of miniature excitatory post-synaptic currents (mEPSCs) of substantia gelatinosa neurons in CFA-induced inflammation via activation of receptor tyrosine kinase and TTX-sensitive (TTX-s) Na^+^ channels. In addition, a previous study demonstrated that acutely applied BDNF enhances the excitability of dissociated small-diameter DRG neurons via activation of the p75 neurotrophin receptor and its downstream sphingomyelin signalling cascade and increases in TTX-R Na^+^ currents [32].

In the present study, we observed that in inflamed rats, the threshold for escape from mechanical stimulation applied to the skin above the MM was significantly reduced two days after CFA injection, suggesting inflammatory hyperalgesia. The present study demonstrated that in dissociated FG/MB-labeled small-diameter TRG neurons; (1) there was a tendency for the BDNF responsive FG/MB-labeled small-diameter TRG neurons to be larger in inflamed rats than naïve rats; (2) the threshold concentration of BDNF that evoked a depolarizing response of neurons in the inflamed rats was significantly lower than in naïve rats; (3) BDNF induced higher frequency and longer lasting spontaneous neuronal discharges in inflamed rats; (4) The relative number of BDNF-induced TTX-R neuronal spikes during current injection was significantly higher in inflamed rats; (5) The BDNF-induced changes in the TTX-R neuron excitability was abolished by K252a. Thus, these findings suggest that BDNF released from small-diameter TRG neurons upregulates trkB through an paracrine/autocrine mechanism, increasing spontaneous activity and triggering the release of chemical neuromodulators (CGRP, SP and ATP) which act on the neighboring neurons or satellite glial cells via a similar mechanism [8-11,53].

BDNF binds to two structurally unrelated plasma membrane receptor types, the p75 neurotrophin receptor and the tyrosine kinase receptor trkB [54]. Activation of trkB by BDNF triggers intracellular signaling cascades that effectively modulate voltage-gated, ligand-gated and second-messenger gated ion channels [52,55]. Cao et al. [8] report that paracrine released BDNF and trkB receptor activation enhanced the excitability of DRG neurons in diabetic neuropathy through voltage-gated A-type potassium channels. In this study we observed that a BDNF-induced depolarizing response was associated with a decrease in cell input resistance. This decreased input resistance in inflamed rats was significantly higher than in naïve rats. The present study shows that BDNF-induced depolarization and increased firing frequency in the TRG neuron excitability was abolished by the tyrosine kinase inhibitor K252a. Since voltage-gated potassium channels determine the resting membrane potential of neurons and regulate their excitability [56], it can be speculated that BDNF-induced increases in the excitability of FG/MB-labeled small-diameter TRG neurons may be due to the depression of voltage-gated potassium currents. This is supported by the following studies; (1) inhibition of voltage-gated potassium currents by somatostatin, interleukin 1β and GDNF in a small-diameter TRG neurons potentiates neuronal excitability, thereby contributing to trigeminal inflammatory hyperalgesia [27,53,57]; (2) BDNF enhances the excitability of small-diameter DRG neurons via activation of p75 neurotrophin receptor and suppression of delayed rectifier-like potassium currents [38]. (3) BDNF significantly affects auditory brainstem neuronal excitability via a decrease in the potassium channels [58]. Although the precise ionic mechanism for BDNF-induced enhanced TRG neuronal excitability is unclear, we hypothesise that BDNF/trkB signaling induces hyperexcitability of the small-diameter TRG neurons via a depression of voltage-gated potassium currents within trigeminal ganglia. Further studies are required to fully elucidate the underlying ion mechanisms between BDNF/trkB signaling and TRG neuronal excitability, including other ion channels.

### Functional significance of BDNF/trkB signaling in the trigeminal ganglia and inflammatory hyperalgesia

BDNF-trkB signaling within the RVM descending pathways has been reported to contribute to the net descending facilitation of synaptic transmission in the Vi/Vc and pain hypersensitivity [18,59]. In addition, inflamed MM primary afferent inputs onto the Vi/Vc region are necessary and sufficient to induce astroglial hyperactivity, concomitant IL-1β induction, and coupling of neuronal NMDA receptor phosphorylation through IL-1β signaling [37]. Thus it can be postulated that modulations of both synaptic transmission in the Vi/Vc and the descending facilitation pathway from the RVM are potential therapeutic targets for the treatment of persisting orofacial pain.

Several reports have demonstrated the functional significance of transganglionic signal communication [8-11,60-62], and we have previously suggested that sensory signals are effectively amplified or attenuated downstream of the ascending sensory pathway and then sent to higher centers. Thus, transganglionic communication, including neuron-satellite glial cell interaction, is one mechanism by which central sensitization can be triggered [53]. We recently provided evidence that in the early stages of CFA inflammation, suppression of the SP/neurokinin-1 receptor mechanism in trigeminal ganglia may prevent central sensitization of hyperexcitability of SpVc wide-dynamic range nociceptive neurons [14]. Taken together, our reports suggest that blocking ganglionic sensitization can effectively reduce the central sensitization of nociceptive neurons and/or neuron-glial interactions in the Vi/Vc transition zone. Therefore, in addition to BDNF/trkB signaling in the central nervous system, the local paracrine mechanism of BDNF/trkB on TRG neurons provides a novel therapeutic target for the treatment of debilitating orofacial deep inflammatory pain.

## Conclusion

The present study provided evidence that BDNF enhances the excitability of the small-diameter TRG neurons projecting onto the Vi/Vc following MM inflammation. These findings suggest that ganglionic BDNF-trkB signaling is a therapeutic target for the treatment of trigeminal inflammatory hyperalgesia.

## Abbreviations

SpVc: Spinal trigeminal nucleus caudalis; C1-2: Upper cervical spinal dorsal horn; Vi/Vc: Trigeminal subnuclei interpolaris/caudalis transition zones; BDNF: Brain-derived neurotrophic factor; trkB: Tyrosine kinase B; TRG: Trigeminal ganglion; MM: Masseter muscle; FG: Fluorogold; MB: Microbeads; IR: Immunoreactive; NMDA: N-methyl-D-aspartate; RVM: Rostral ventromedial medulla; ATP: Adenosine triphosphate; SP: Substance P; GDNF: Glial-derived neurotrophic factor; DRG: Dorsal root ganglion; CFA: Freund’s adjuvant; PBS: Phosphate buffered saline; EDTA: Ethylene diaminetetra acetic acid; HEPES: N-2-hydroxyethylpiperazine-N’-2-ethanesulfonic acid; EGTA: Ethylene glycol-bis-β-aminoethyl ether N,N,N’,N’-tetra acetic acid; TTX: Tetrodotoxin; TTX-s: TTX-sensitive; TTX-r: TTX-resistant; ANOVA: Analysis of variance; trkA: Tyrosine kinase A; CGRP: Calcitonin gene related peptide; TNFα: Tumor necrosis factor α; IL-1β: Interleukin 1β; MAP: Mitogen-activated protein kinase; mEPSCs: Miniature excitatory post-synaptic currents.

## Competing interests

The authors declare that they have no competing interests.

## Authors’ contributions

MT (CA) participated in the design of study, carried out the experiment and wrote the manuscript. MT and JK carried out retrograde-labeling and the electrophysiological experiment. TK and MN carried out the immunohistochemical experiment. SM provided data interpretation and helped to finalize the manuscript. All authors read and approved the final manuscript.
